# UPS 2.0: unique probe selector for probe design and oligonucleotide microarrays at the pangenomic/ genomic level

**DOI:** 10.1186/1471-2164-11-S4-S6

**Published:** 2010-12-02

**Authors:** Shu-Hwa Chen, Chen-Zen Lo, Sheng-Yao Su, Bao-Han Kuo, Chao A Hsiung, Chung-Yen Lin

**Affiliations:** 1 Institute of Information Science, Academia Sinica, No. 128 Academia Rd., Sec. 2, Taipei 115, Taiwan; 2 Division of Biostatistics and Bioinformatics, National Health Research Institutes. No. 35 Keyan Rd. Zhunan, Miaoli County 350, Taiwan; 3 Institute of Fisheries Science, College of Life Science, National Taiwan University, No. 1, Roosevelt Rd. Sec 4, Taipei, Taiwan; 4 Research Center of Information Technology Innovation, Academia Sinica, No. 128 Yan-Chiu-Yuan Rd., Sec. 2, Taipei 115, Taiwan

## Abstract

**Background:**

Nucleic acid hybridization is an extensively adopted principle in biomedical research, in which the performance of any hybridization-based method depends on the specificity of probes to their targets. To determine the optimal probe(s) for detecting target(s) from a sample cocktail, we developed a novel algorithm, which has been implemented into a web platform for probe designing. This probe design workflow is now upgraded to satisfy experiments that require a probe designing tool to take the increasing volume of sequence datasets.

**Results:**

Algorithms and probe parameters applied in UPS 2.0 include GC content, the secondary structure, melting temperature (Tm), the stability of the probe-target duplex estimated by the thermodynamic model, sequence complexity, similarity of probes to non-target sequences, and other empirical parameters used in the laboratory. Several probe background options,***Unique probe within a group****,****Unique probe in a specific Unigene set****,****Unique probe based onthe pangenomic level****,* and ***Unique Probe in the user-defined genome/transcriptome****,* are available to meet the scenarios that the experiments will be conducted. Parameters, such as salt concentration and the lower-bound Tm of probes, are available for users to optimize their probe design query. Output files are available for download on the result page. Probes designed by the UPS algorithm are suitable for generating microarrays, and the performance of UPS-designed probes has been validated by experiments.

**Conclusions:**

The UPS 2.0 evaluates probe-to-target hybridization under a user-defined condition to ensure high-performance hybridization with minimal chance of non-specific binding at the pangenomic and genomic levels. The UPS algorithm mimics the target/non-target mixture in an experiment and is very useful in developing diagnostic kits and microarrays. The UPS 2.0 website has had more than 1,300 visits and 360,000 sequences performed the probe designing task in the last 30 months. It is freely accessible at http://array.iis.sinica.edu.tw/ups/.

Screen cast: http://array.iis.sinica.edu.tw/ups/demo/demo.htm

## Background

Nucleic acid hybridization is an extensively adopted principle in biomedical research. Several methods have been derived based on this principle to detect targets using sequence-specific probes (*e.g.*, northern blot, southern blot, and *in situ* hybridization). The lab protocol for probe hybridization is further optimized and miniaturized into microarray format to detect transcriptional activity of thousands of genes simultaneously [1]. The performance of these widely adopted methods depends on the specificity of probes to their targets. The selection of suitable oligonucleotide probes remains a bottleneck in the microarray workflow [2]. To find the best, unique probe(s) for detecting target(s) from a sample cocktail, we developed an algorithm and implemented this algorithm in a probe design web platform, the Unique Probe Selector (UPS) [3]. The parameters used in UPS include GC content, GC clamps, the duplex stability estimated by thermodynamic theory model, the secondary structure of probes, a low-complexity mask, and other empirical preferences of wet-lab researchers. Thus, this probe design tool is able to overcome the problem of background noise during hybridization.

Several tools are available for probe design. OligoArray 2.1 [4,5], GoArrays [6], OligoPicker [7], ArrayOligoSelector [8], YODA [9], ProbeSelect [10], and Picky [11] are stand-alone tools that run in command mode and provide unique probe candidates for input sequences under the constraints of GC%, Tm, absence of low complexity, and position near the 3' end. OligoWiz2 [12] is a java-based server-client solution for probe design in a graphical user interface. Oligodb [13] and OligoArrayDb [14] are databases providing pre-calculated probes for human/mouse transcriptome and sequenced Archaea/Bacteria/Eukaryote species, respectively. Golfier *et al*. [15] developed an informatics tool called SOL, which updates mouse sequences in the NCBI/RefSeq semi-automatically and outputs a collection of mouse oligonucleotide probe candidates. Researchers can set up an SOL and refine the parameters when working on probe design tasks for other species. Although most of these tools are useful when designing nucleotide probes, few consider background noise of cross-hybridization, or require user skills to set up and optimize the tool for an experiment. Thus, we implemented a user-friendly web-based tool UPS [3] for designing probes with small likelihoods of forming non-specific duplexes to the other submitted targets in the same query batch or to the sequence set of an NCBI/Unigene-listed species, and upgrades its capability for a customized reference sequence set in the current version.

Recent advances in sequencing methods, such as next generation sequencing (NGS) technology, allow researchers to decrypt gene contexts of an unknown genome faster and cheaper than ever before. These methods run millions of sequencing reactions in parallel, generating short sequence reads on the gigabase-scale, and harvest assembled contigs by algorithms. These methods have been successfully applied for genome re-sequencing and have been expended to resolve novel genomes and multiple genomes in a mixture (the approach of metagenome). An application for sequencing a transcriptome to resolve the gene context of an unknown genome is also available. Since both the time and cost of sequencing a transcriptome or genome are markedly reduced, researchers are willing to use a sequencing approach to leap over the bottlenecks when conducting a classical genome/EST library sequencing project. Thus, the collection of non-target sequences of a hybridization experiment has an increased possibility of its availability, even when the target organism is not a well-studied model species. The NGS assemblies can also be raw material for designing probes for fabricating arrays such as 16S rRNA gene-based diagnostic arrays [16]. Several widely used tools, such as ORMA [17], ARB [18], and PRIMROSE [19], are available for classification and phylogenetic analysis of bacterial species. These tools conduct the probe design process when the collection of molecular phylogenetic markers (*i.e.*, 16S rRNA) is updated along with the renewal of the sequence database. The primer design strategy of each tools are different; it is difficult in choosing probes provided from different tools.

**Figure 1:**
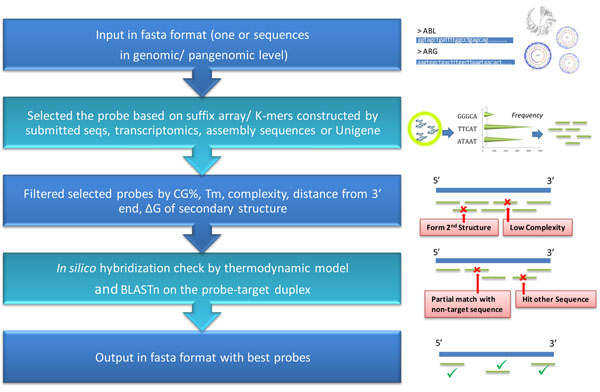
The concept of UPS 2.0 for pangenomic/genomic studies.

**Figure 2:**
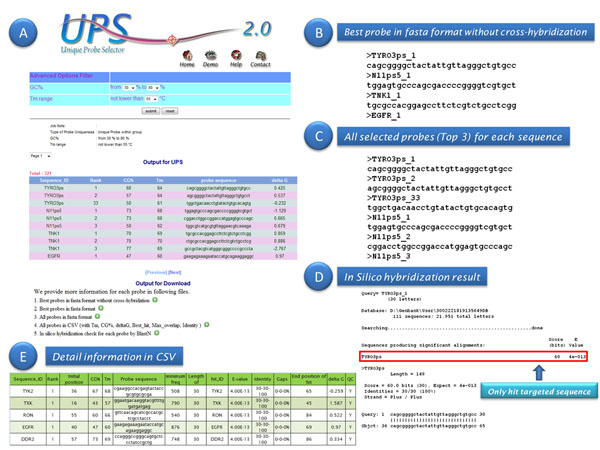
Output of UPS 2.0. (A) The advanced filter by Tm and GC%, and the probe list with downloadable files. (B) The output for the best probe without cross-hybridization in FASTA format. (C) All selected probes (Top 3) for each sequence. (D) *In silico* hybridization result based on blastn with several checking rules. (E) Detailed information in the tabular format.

As mentioned, the UPS algorithm can find best probes for input target sequences using parameters of sequence uniqueness and duplex stability to achieve good hybridization uniformity (Fig. 1). In UPS 2.0, two new options have been added for probe design, and the reference sequence database and server hardware framework have been upgraded. The new option “Unique Probe based on the pangenomic level” allows users to employ environmental nucleotide sequences (Env_NT) or the non-redundant NCBI nucleotides database (NCBI_NT) as a potential background noise source. Probe designed using this option can be applied in detecting sequences from samples with mixed genomes or transcriptomes such as specimens in a metagenomic study. Another new function, “Unique Probe in the user-defined genome/ transcriptome,” selects probes for target sequences using the genome/transcriptome sequence set (in FASTA format) as the background reference uploaded with the probe designing query. The background reference in the option “Unique probe in the specific Unigene set” is updated; currently, 128 species derived from Unigene (Feb 2010) are listed in this option. Some probe parameters, such as the number of probe candidates for each submitted sequence, GC%, salt concentration, and lower-bound value of probe melting temperature, can be adjusted for increased flexibility of UPS pipeline. Besides, one tendency in the post-genomic era is the fast growth of sequences deposited in public sequence databases or resolved in an individual lab, thus the increased target sequence entries will be submitted in a probe design query. Therefore, an automatic mechanism to check the redundancy of sequence identifier (ID) in input file is essential for avoiding the chaos in probe ID assignment. The outputs of UPS 2.0 are presented in several layout formats, such that output fits the special needs of users (Fig. 2).

## Results

### Criteria for probe selection in UPS 2.0

To reduce the likelihood of non-target hybridization, we designed a workflow for finding the best probes of input sequences based on uniqueness of probe sequence and probe-to-target duplex stability. The Nearest-Neighbor model [20] and blastn were applied to identify probe candidates for each input sequence with a minimal likelihood of cross-hybridization. First, probes with single, di-, or tri- nucleotide repeats are excluded as described previously. Next, the similarity of probes for a non-target sequence is evaluated. The following criteria developed by Li *et al.*[21] are used to further exclude unsuitable oligonucleotides: 1) overall similarity of a probe to its non-target sequences in the same submitted batch should be <85%; and, 2) a continuous identical stretch of a probe to non-target sequences must not exceed 17 bases. Both criteria integrated with blastn are called *in silico* hybridization in this work. Furthermore, the uniqueness of probes is assessed using the frequency table built from the reference sequence set given in one of three customized options. The simplest condition is finding unique probes for each sequence among an input sequence set (the “***Unique probe within a group***” option). The second option, “***Unique probe in the specific Unigene*,**” is for devising unique probes for each submitted sequence with the smallest chance of cross-hybridizing the selected Unigene set. To meet the needs of researchers conducting metagenomic studies, this work developed a new option, "***Unique Probe based on the pangenomic level***", which allows users to design probes using the environmental nucleotide sequences (Env_NT) or the non-redundant NCBI nucleotides database (NCBI_NT) as background reference set. Via another novel option, “***Unique Probe in the user-defined organism*,**” users can design probes by adopting uploaded sequences as the background reference. The file size of the uploaded reference sequence set can be up to 30 Mb, and the sequence length of a single sequence entry is up to 5 million bases. Probe candidates passing the criteria (*e.g.,* Tm cutoff, GC%, the proximity of 3' end, *in silico* hybridization (sequence complexity and homology to non-target sequence), are ranked by the E-value of the probe-target duplex (blastn) using the scoring matrix built from the customized reference sequence. Additional details of *in silico* hybridization can be found on the UPS 2.0 online HELP page (http://array.iis.sinica.edu.tw/ups/help1.html).

### Applications and experimental validation

#### Scenario 1. Best probes for detecting specific targets from PCR fragments

Using common primer sets, such as a consensus primer set of small subunit rRNA gene region, a bundle of PCR products can be amplified from a sample with mixed microbial genomes. To detect the presence of a particular species, a dot blotting assay can be contrived based on a small set of probes with good discrimination power. Using the UPS option “Unique Probe within a group,” a user can find unique probes for each sequence with minimal likelihood of matching non-target sequences in the uploaded sequence set. Such a probe set is suitable for detecting malicious pathogens in environmental samples or food contaminants. If target sequences are mixed with various genomes, such as sludges from sanitary sewers, one can use the option “Unique Probe based on the pangenomic level” to assign all known environmental sequences as the reference background. The assay can be started with a PCR using degenerated primer sets to amplify target sequences, following by an hybridization on a small array with sequence-specific probes to detect the presence of specific bacteria species.

#### Scenario 2. Finding probes to detect exogenous/pathogenous genes on a Unigene-listed species

The spatiotemporal activity of a pathogenic genome is a critical issue. To design probes for detecting pathogen sequences in a specimen from a particular organism in the Unigene species list (*e.g.,* human) or a closely related one, we may set pathogen sequences as the input set in the option “Unique Probe in a specific Unigene set” and assign the sequence set of the species (Homo_sapiens, in this case) as the reference background. Then, UPS 2.0 will find suitable probes for identifying pathogen sequences with low false-positive hits on host sequences.

#### Scenario 3. Unique probes working on the user-uploaded sequence(s) as the reference background

Unigene collects EST clusters from more than 100 species; however, partially or unpublished sequence assemblies from species other than those in Unigene list may be available in individual labs. To design probes working in a biopsy from a particular organism, such as detecting pathogen transcripts, one can assign the background by uploading a set of reference sequences (a file in FASTA format) of this species using the option “Unique Probe in the user-defined organism.” Thus, pathogen-specific probes will be selected using UPS criteria, which ensure reduced likelihood of cross-hybridization of a probe to a non-target sequence.

### Verification by experiment

**Figure 3:**
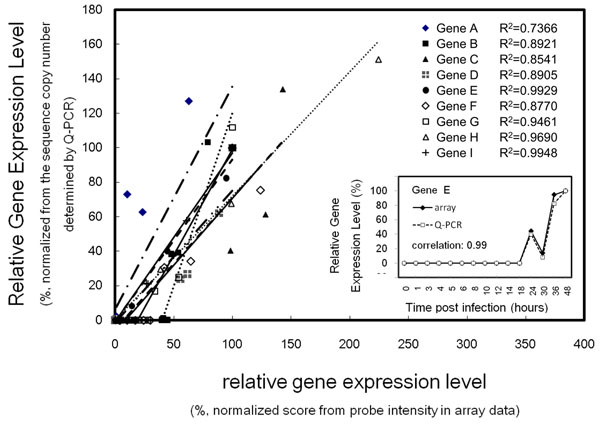
Validation of the performance of UPS 2.0 designed probes by QPCR. The expression level of nine white spot syndrome virus (WSSV) genes, determined by either QPCR or a microarray, were normalized to the measurement at 48h post viral infection. The coefficients of linear regression of gene-to-probe pairs are >0.7. The gene profile of an example, gene E, is plotted in the insert panel. The normalized measurement by QPCR and the microarray are strongly correlated for gene E (Pearson’s correlation coefficient, 0.99) and all others in this test (data not shown).

A cooperation project with researchers devoted to shrimp virology is ongoing. The researchers carried out intensive sequencing tasks for deciphering the shrimp virus (WSSV) genome [22] and shrimp transcriptome [23]. The primary goal of this project is to track the expression of viral genes throughout the entire infection process. Here, a new strategy in UPS 2.0 is utilized to design probes for a shrimp/pathogen microarray. Briefly, EST reads from *Penaeus monodon* (roughly 25,000 sequences, public database + unpublished data) are assembled into roughly 7,000 clusters (contigs). Together with 701 putative viral (ORFs), these sequences were submitted to UPS 2.0 via the option “*Unique Probe within a group*” to produce customized microarrays in the 44k format (six probe replicates for each submitted sequence) by *in situ* synthesis technology (Agilent). The mRNA was extracted from the time-coursed samples (infected shrimp tissue, gills in this case, 0–48 hours) from viral challenge experiments and the gene profile was sketched using the microarray platform. The researchers randomly picked several viral ORFs to assess the correlation between array data and QPCR data (Fig. 3). In most cases, the correlation between gene expression levels quantified by the array and by QPCR is strong, as evaluated by the correlation between two expression profiles (Pearson’s correlation coefficient) and the linear correlation R^2^ of each probe/gene pair. The coefficients of linear regression of gene-to-probe pairs were >0.7 and were strongly correlated (Pearson’s correlation coefficient, 0.86–0.99).

## Discussion and conclusions

UPS 2.0 is an updated from the previous platform, which is a probe design tool for identifying the unique segment in each submitted sequence in a query dataset, or the unique segment of input sequences in the user-defined background. With the flexible and intuitive interface in UPS 2.0, users can easily customize their probe design parameters by following the user-friendly online instruction and obtain probes with quality information. Probes designed in UPS 2.0 can be utilized to detect a target in a classical hybridization-based assay. Notably, probes from such batch-wise query meet the requirements of microarray probe design. In UPS 2.0, Unigene data are updated, and UPS 2.0 can take the user-uploaded sequence set as a background reference. To date, more than 100 species are listed in the current Unigene dataset and this number continues to increase. The new option allows users to upload a reference set other than the Unigene list. These improvements expand the choice of a user-defined reference during probe design.

The UPS workflow mimics the condition of target/non-target mixture in an actual experiment to be carried out in a lab bench. This is beneficial when developing a kit for molecular diagnosis or typing. Thus, probes designed by UPS 2.0 are particularly suitable for detecting infectious agents, such as bacteria and viruses, with little chance of cross hybridization of host transcripts, or for detecting a specially selected gene list (gene signatures) using small arrays with minimal background noise. A single entry in the user-uploaded reference set can be 5×10^6^ bps, meaning that most prokaryote genomes can serve as the working probe background in UPS 2.0.

UPS has run stably for over 2 years. More than 1,300 visitors have submitted their queries and about 360,000 sequences have been submitted to UPS 2.0. The performance of UPS will be continually improved by applying a relatively more sophisticated algorithm and reducing calculation time. The spectrum of reference backgrounds has been expanded by incorporating published metagenomic datasets, environmental nr, and the entire genome sequence set in the public domain into customized options to optimize oligonucleotide probe design for experiments on the pangenomic/genome scale.

## Methods

### System implementation

To improve the performance of UPS 2.0, the hardware was upgraded on symmetrical multi-processor (SMP) PCs equipped with quad CPUs (Intel Xeon E5420 2.5GHz) and 16 GB RAM. The LAPP structure (SUSE Linux Enterprise Server 10, Apache v.2.04, PostgreSQL v.8.2.4, PHP v.5.1.0) facilitates web access, file uploading, format checking, result storing/downloading, and job controlling, and supports e-mail notifying functions.

All calculations in the core algorithm for suffix arrays, constraints for probe selection (GC%, low complexity masking, distance from the 3' end), free energy for a probe’s secondary structure, melting temperature estimated in a thermodynamic model, and *in silico* hybridization based on the NCBI blastn [20] with several checking rules, *i.e*., percentage of similarity and maximum overlapped bases of continued sequences between probe and possible targets, are performed in Boland Delphi 2006 on an MS Windows 2003 server. To provide the most updated background reference, an automatic procedure for rebuilding suffix arrays for each species from the updated Unigene databases is currently scheduled.

### Hybridization parameters

UPS parameters were described in Chen *et al*[3]. Some probe parameters are adjustable with increased flexibility in UPS 2.0 in response to user requests. Briefly, probe length is adjustable (range, 30 - 120 mers in length). The salt concentration is set at 0.58M and is adjustable (range 0 - 1M) to fit the ionic strength in the user-defined condition. Probe candidates with a low complexity segment(s), such as five or more continual nucleotides and continuous di-/tri-nucleotide repeats, are excluded. The acceptable GC% of probes is 30 - 70% empirically and can be adjusted by users. Further constraints on probe features, GC% and the lower-bound of Tm, are available in the output browsing interface. The melting temperature of probes is calculated based on the Nearest-Neighbor model [21], and the secondary structure of a designed probe is calculated via the Perl program UNAFold.pl [24]. Thermodynamic theory was integrated to estimate the ΔG of probe-target duplexes, probe-non-target duplexes, and the probe secondary structure.

## Competing interests

The authors declare that they have no competing interests.

## Authors' contributions

SHC and CYL designed the algorithm and method, and drafted the manuscript together. CZL, SYS and BHK were responsible for implementation and system integration. SHC and CAH participated in discussions and conceptualization as well as revising the draft. All the authors read and approved the manuscript.
